# Academic self-efficacy, grit, and teacher support as predictors of psychological well-being of Chinese EFL students

**DOI:** 10.3389/fpsyg.2023.1332909

**Published:** 2024-01-08

**Authors:** Lin Tang, Xiaojing Zhu

**Affiliations:** ^1^School of Foreign Languages, Shandong University, Jinan, China; ^2^School of Public Education, Shandong University of Arts, Jinan, China

**Keywords:** academic self-efficacy, grit, teacher support, psychological well-being, Chinese EFL students, structural equation modeling (SEM)

## Abstract

**Introduction:**

This study explores the determinants of psychological well-being among 968 Chinese English as a Foreign Language (EFL) students by employing structural equation modeling (SEM). Focusing on academic self-efficacy, grit, and teacher support, this research aims to illuminate their roles in shaping the psychological well-being of EFL students within the Chinese educational context.

**Methods:**

Data from a robust sample of Chinese EFL students were analyzed using SEM techniques to investigate the relationships between academic self-efficacy, grit, teacher support, and psychological well-being. Validated instruments were utilized to measure these constructs, ensuring accuracy and reliability. The study employed meticulous data collection procedures over a three-month period, maintaining strict ethical standards and anonymity for participants.

**Results:**

The SEM analysis revealed intricate connections among academic self-efficacy, grit, teacher support, and the psychological well-being of Chinese EFL students. Academic self-efficacy and grit emerged as direct predictors of psychological well-being, highlighting their significance in fostering students’ overall well-being. Additionally, teacher support was identified to play a mediating role in this relationship, emphasizing its critical influence on enhancing academic self-efficacy and grit, thereby contributing to students’ psychological well-being.

**Discussion:**

These findings underscore the crucial importance of nurturing academic self-efficacy and grit to enhance the psychological well-being of Chinese EFL students. Furthermore, teacher support stands out as a pivotal factor in elevating students’ academic self-efficacy and grit, emphasizing the need for educational interventions centered on fostering these attributes among Chinese EFL learners. The implications of these results extend to educational practices, emphasizing the indispensable role of teacher support and interventions aimed at cultivating academic self-efficacy and grit to promote the psychological well-being of EFL students within the learning environment.

## 1 Introduction

Academic success and psychological well-being are two vital elements of learners’ educational experiences. In the context of Chinese English as a Foreign Language (EFL) education, exploring the factors that affect students’ psychological well-being is of utmost importance. Students’ psychological well-being not only influences their overall satisfaction and adjustment to the learning environment but also has implications for their academic achievements (Virtanen et al., 2019; Cobo-Rendón et al., 2020; Zhou, 2023). This study embarks on a comprehensive investigation to shed light on the predictors of psychological well-being among Chinese EFL students.

One of the key determinants under scrutiny in this research is academic self-efficacy, a concept firmly rooted in Bandura’s (1986) social cognitive theory. Academic self-efficacy pertains to students’ beliefs in their capabilities to perform tasks and achieve desired outcomes in the academic domain (Diseth et al., 2012; Hoigaard et al., 2015). A robust body of research has consistently demonstrated that academic self-efficacy is positively associated with academic performance and psychological well-being (García-Álvarez et al., 2021; Matteucci and Soncini, 2021). However, within the Chinese EFL context, the specific role of academic self-efficacy in shaping psychological well-being remains an open question.

Grit, a relatively newer construct, has gained prominence in the educational psychology literature (Duckworth et al., 2007). Grit is defined as a combination of passion and perseverance for long-term goals (Duckworth and Gross, 2014; Zhao and Wang, 2023). It represents a student’s ability to persist in the face of challenges and setbacks, which is particularly relevant in the demanding and often challenging landscape of EFL learning (Duckworth and Quinn, 2009; Teimouri et al., 2022). Grit has shown promise in predicting academic success and psychological well-being in various educational settings (Eskreis-Winkler et al., 2014; Duckworth, 2016). However, the extent to which grit contributes to the psychological well-being of EFL students remains a topic requiring further exploration.

Teacher support, an influential factor in students’ academic experiences, also merits attention. A supportive teacher-student relationship has been linked to enhanced academic engagement, motivation, and overall satisfaction (Aldridge et al., 2013; Brandseth et al., 2019; Tao et al., 2022). In the context of EFL education, where students confront distinct hurdles like linguistic obstacles and variances in culture, the significance of educator assistance in influencing emotional health garners specific attention (Virtanen et al., 2019; Neufeld and Malin, 2020).

This study employs structural equation modeling (SEM) to investigate the complex relationships among academic self-efficacy, grit, teacher support, and the psychological well-being of EFL students. Through the exploration of these relationships, we aim to provide valuable insights for educators and policymakers to enhance the educational experience of Chinese EFL students. By understanding the specific contributions of academic self-efficacy, grit, and teacher support to psychological well-being, this research seeks to inform targeted interventions and practices that can lead to improved academic success and overall well-being within the EFL context.

## 2 Review of literature

### 2.1 Psychological well-being

Psychological well-being encompasses a multifaceted blend of emotional states and effective functioning in personal and social domains (Ryff, 2014). This construct is characterized by a subjective sense of contentment, including facets such as happiness, life satisfaction, and a sense of fulfillment in various life roles (Ryff and Keyes, 1995). It is important to note that psychological well-being is a dynamic concept, ranging from negative mental states like anxiety and emotional strain to a state characterized by positive mental health (Diener, 1984; Warr, 2013; Sagone et al., 2023). Bornstein et al. (2003) expounded on well-being as an enduring state of successful performance across the lifespan, integrating physical, cognitive, and socio-emotional functions. This state is reflected in the ability to cultivate fulfilling social relationships and effectively navigate moderate psychosocial and environmental challenges. The conceptualization of well-being delineates two fundamental dimensions: subjective well-being (SWB) and psychological well-being (PWB) (Ryff, 1989; Ryff and Singer, 2008; Lin, 2015; Villarosa and Ganotice, 2018).

Subjective well-being, aligned with the hedonic perspective, encompasses facets related to physical health, positive and negative affect, and overall life satisfaction (Ryff and Singer, 2008). Conversely, psychological well-being, grounded in the eudaimonic perspective, encompasses dimensions such as self-actualization, personal development, and harmonious relations with the environment (Ryff, 1989; Keyes et al., 2002). Ryff and Keyes (1995) expound on the six dimensions of psychological well-being, each posing distinct challenges in fostering positive functioning. These dimensions entail the pursuit of self-acceptance, the cultivation of warm and trusting interpersonal relationships, adeptly managing one’s environment to meet personal needs and aspirations, seeking self-determination and personal authority, deriving purpose and meaning from one’s endeavors, and endeavoring to unfold one’s inherent talents and capacities.

Psychological well-being has garnered considerable attention within educational contexts, not only concerning the well-being of learners but also increasingly focusing on the psychological health and well-being of educators, acknowledging its pivotal role in fostering conducive learning environments (Gustems-Carnicer and Calderón, 2013; Hanson et al., 2016; Weiss et al., 2016; Xiyun et al., 2022; Zhang et al., 2023). When investigating the psychological well-being of college and university students, numerous research endeavors have delved into a range of influential factors (Greenier et al., 2021). For instance, Flett et al. (2019) underscored the significance of the concept of “mattering” and its pronounced connection with students’ psychological well-being. Their recommendations lean towards the implementation of campus-based initiatives aimed at augmenting students’ sense of mattering, ultimately contributing to the enhancement of their psychological well-being. Furthermore, Bano et al. (2019) directed their focus toward the examination of the impact of WhatsApp usage on the psychological well-being of students. Their investigation shed light on the intermediary functions played by social capital and social integration in this particular association. Their study highlights the imperative need to take into account these contributing factors when evaluating the consequences of employing digital communication platforms on students’ overall well-being.

Meanwhile, Liu et al. (2019) delved into the dynamics of changes in the psychological well-being of undergraduate students as they transitioned to university life. Their empirical findings suggested a nuanced impact during this critical transitional phase, encompassing both positive and negative effects on students’ psychological well-being. In response to these findings, the researchers recommended the development of targeted interventions and support mechanisms to assist students in successfully navigating this pivotal life transition. In summary, these studies collectively underscore the multidimensional nature of factors influencing the psychological well-being of college and university students.

### 2.2 Academic self-efficacy

Within the framework of Bandura’s (1986) social cognitive theory, academic self-efficacy reflects an individual’s belief in their ability to effectively navigate and excel in academic pursuits. This construct encompasses the confidence required to adeptly handle academic tasks, engage with educational materials, and attain desired learning outcomes (Pajares, 1996). It serves as a fundamental motivational driver, guiding students’ efforts and sustaining their determination to achieve academic success (Zimmerman, 2000).

It is worth noting that academic self-efficacy contextualizes the confidence students hold specifically in their academic abilities (Multon et al., 1991). It revolves around their belief in effectively mastering academic challenges, completing tasks, and realizing educational goals. This cognitive assessment plays a pivotal role in shaping students’ behaviors, influencing their commitment to learning, and shaping their academic achievements (García-Álvarez et al., 2021). In educational research, the academic self-efficacy of students has become a focal point of interest. Previous studies have consistently shown a positive association between academic self-efficacy and a range of favorable academic outcomes, including improved academic performance and increased engagement in the learning process (Multon et al., 1991; Honicke and Broadbent, 2016; Huang, 2016; Sagone and Indiana, 2023). Additionally, a robust sense of academic self-efficacy has been recognized as a protective factor against the adverse impacts of academic stressors experienced by students (Ye et al., 2018). This construct helps students navigate challenges and maintain a positive attitude toward their academic pursuits, thus contributing significantly to their psychological resilience in the face of academic pressures (Salami, 2010).

In the context of this study, the exploration of academic self-efficacy is particularly relevant due to its potential implications for the psychological well-being of students (Siddiqui, 2015; Cobo-Rendón et al., 2020; Fathi et al., 2023). Individuals with high levels of academic self-efficacy often exhibit reduced anxiety and stress levels in academic settings, creating an environment conducive to enhanced well-being (Ouweneel et al., 2013). Moreover, these individuals, armed with unwavering confidence in their academic abilities, approach challenges with a proactive, problem-solving orientation, nurturing an overall sense of well-being (Schwarzer and Jerusalem, 1995). It is essential to acknowledge that academic self-efficacy is a multifaceted construct influenced by a variety of factors. Cultural norms, societal expectations, personal experiences, feedback from educators, and interactions with peers all contribute to the formation and perpetuation of academic self-efficacy beliefs (Usher and Pajares, 2008).

Numerous investigations have explored the link between academic self-efficacy and psychological well-being across different settings. Siddiqui (2015) examined how self-efficacy influences the psychological well-being of undergraduate students. Expanding on this, Matteucci and Soncini (2021) extended their inquiry to include Italian university students, including those dealing with Specific Learning Disorder, and investigated the role of self-efficacy in shaping their psychological well-being. Salami (2010) delved into the realms of emotional intelligence, self-efficacy, and psychological well-being, elucidating their interplay and the implications for the quality of education.

In another study, García-Álvarez et al. (2021) scrutinized the interrelationship between emotional intelligence, academic self-efficacy, and the well-being of college students in Venezuela, offering valuable insights into the dynamics of these factors during times of crisis. Costa et al. (2013) conducted a study examining emotional intelligence and self-efficacy in college students, highlighting their impact on psychological well-being. In another significant investigation, Hong et al. (2022) explored the mediating effects of self-efficacy and resilience on the psychological well-being of the Lebanese population amidst the concurrent crises posed by the COVID-19 pandemic. This study illuminated the intricate interplay of self-efficacy and resilience in shaping psychological well-being during times of adversity. These collective efforts significantly enrich our understanding of the intricate relationship between academic self-efficacy and psychological well-being in diverse populations and contexts.

In summary, academic self-efficacy serves as a critical determinant of both academic achievement and psychological well-being. For Chinese EFL students, understanding the intricate interplay of factors shaping academic self-efficacy can provide valuable insights into the elements influencing their overall psychological health. These insights are invaluable and offer a foundation for tailored educational interventions aimed at enhancing their academic process.

### 2.3 Grit

Grit, characterized by sustained effort and unwavering commitment to one’s goals, stands as a foundational determinant of success, enabling individuals to persist in the face of obstacles and setbacks (Duckworth et al., 2007). This enduring trait is closely associated with the capacity to sustain interest, curiosity, and passion over extended periods while striving to achieve objectives, regardless of the barriers encountered (Duckworth, 2016; Alamer, 2021; Lan et al., 2021). Research consistently underscores the significance of grit in diverse domains, including the acquisition of new skills, such as language learning (Duckworth, 2016; Lee, 2022; Teimouri et al., 2022; Fathi and Hejazi, 2023).

Numerous studies have also highlighted the positive correlation between grit and various aspects of academic performance, scholastic achievements, self-confidence, engagement, and motivation (Akos and Kretchmar, 2017; Hodge et al., 2018). However, it is worth noting that some research findings have failed to establish a robust link between grit and academic success (Ivcevic and Brackett, 2014). The inconclusive results in this regard may be attributed to several moderating factors, especially the nature of the task or domain. It is suggested that more challenging and well-defined tasks may require a higher level of grit, potentially influencing its impact on easier tasks differently (Credé et al., 2017). Furthermore, the relationship between grit and performance may be influenced by individual differences, such as inherent abilities and metacognitive skills. High levels of grit may not yield the desired outcomes if self-regulation and aptitude are lacking. In some cases, excessive grit might even discourage individuals from seeking help, which has been shown to be beneficial for achievement (Alamer, 2021; Khajavy et al., 2021; Teimouri et al., 2022).

In the context of second language learning, Wei et al. (2019) reported both direct and indirect associations between grit and foreign language performance. Similarly, in a study by Feng and Papi (2020), perseverance of effort was identified as a predictor of L2 persistence and motivation. Numerous investigations have delved into the intricate dynamics between grit and psychological well-being, offering valuable insights into this pivotal connection. Vinothkumar and Prasad (2016) explored the pivotal role of resilience as a moderating factor in the association between grit and psychological well-being, enriching our comprehension by revealing how resilience can intricately shape the impact of grit on overall well-being. Arya and Lal (2018) took a comprehensive approach, examining grit and the sense of coherence as predictors of well-being. Their study underscored the multifaceted nature of grit’s influence on psychological well-being, emphasizing the significant role that a sense of coherence plays in understanding how grit contributes to one’s overall state of well-being. In a similar vein, Vainio and Daukantaitė (2016) explored the nexus between grit and various dimensions of well-being, unveiling both direct and indirect relationships. They illuminated the intricate pathways through which grit can affect different aspects of well-being, involving variables like the sense of coherence and authenticity. Furthermore, in a recent study, Huo (2022) investigated the reciprocal relationship between learners’ psychological well-being and academic engagement in shaping their grit. This research highlighted the dynamic feedback loop where learners’ well-being can, in turn, influence their level of grit, reinforcing the interconnectedness of these constructs.

Despite the valuable insights offered by these studies, the variability in findings across different investigations underscores the need for further research to enhance our understanding of grit, particularly within the context of EFL learning. This extended inquiry aims to clarify whether the concept of grit can be effectively applied to the domain of language learning, where persistence through a lengthy process marked by errors and challenges is often required.

### 2.4 Perceived teacher support

Perceived teacher support constitutes a cornerstone of the educational experience, intricately intertwined with students’ motivation, engagement, and overall well-being (Cox and Williams, 2008; Metheny et al., 2008). It encompasses students’ perceptions of their teachers’ responsiveness, care, and availability to offer guidance when needed (Mercer et al., 2011). This element is particularly pertinent for EFL students, who often encounter linguistic and cultural hurdles in their academic journey.

In the realm of emotional support, the notion is categorized into general and specific categories. Specific emotional support, including parental support and teacher support, falls under the umbrella of social support (Yasin and Dzulkifli, 2010; Lei et al., 2018). Teachers can offer students both practical and emotional support by providing information, appraisal, and instrumental help (Liu et al., 2021). Teacher emotional support plays a pivotal role in students’ lives, influencing their psychological well-being. It fosters trust, care, love, respect, and rapport in the classroom environment, alleviating students’ anxiety, loneliness, and depression (Yang et al., 2018; Longobardi et al., 2019). Thus, teacher emotional support acts as a buffer against students’ anxiety and can effectively manage psychological problems (Romano et al., 2020).

Research consistently highlights the transformative impact of supportive and empathetic teachers on the academic and psychological well-being of students. If learners consider their instructors as approachable and understanding, they are more likely to experience a profound sense of belonging within the classroom environment (Wang, 2012). This sense of belonging is closely associated with heightened academic engagement and positive emotional experiences, significantly contributing to their overall psychological well-being (Walton and Cohen, 2007; Mohammad Hosseini et al., 2022). Moreover, perceived teacher support has a positive influence on students’ self-efficacy beliefs. When students recognize that their teachers are invested in their success, it bolsters their belief in their own capabilities to achieve their educational goals (Wentzel, 1998; Derakhshan et al., 2022). As discussed earlier, academic self-efficacy is a potent predictor of both academic success and psychological well-being, underscoring the far-reaching implications of teacher support on students’ overall well-being.

Perceived teacher support comprises both emotional and instructional dimensions, with academic and emotional support serving as primary facets (Miller et al., 2000; Longobardi et al., 2016). It forms an integral part of the teacher-student relationship and is consistently associated with students’ academic emotions, engagement, and achievement (Ryan et al., 2005; Spilt et al., 2011; Roorda et al., 2017). The perceived supportiveness of teachers creates an environment where students feel more inclined to participate actively in classroom activities, ultimately contributing to a positive learning atmosphere (Sadoughi and Hejazi, 2021; Derakhshan et al., 2023). Furthermore, the quality of teacher support significantly influences students’ engagement and effort in their studies (Ansong et al., 2017). This support has a direct, positive impact on students’ active involvement in their learning experiences (Sadoughi and Hejazi, 2021). Nevertheless, it is crucial to note that although some studies affirm this direct link, others suggest that the association between perceived teacher support and learning engagement warrants further investigation (Wang and Zhang, 2020).

Teacher support’s influence extends to academic achievement, with learners’ perceptions of their teachers’ support consistently associated with their educational success (Vollet et al., 2017). The care and encouragement that teachers provide on a daily basis are equally crucial in promoting academic progress, demonstrating the multifaceted role of teacher support in shaping students’ academic outcomes and overall well-being (Tao et al., 2022). Numerous inquiries have explored the nexus between teacher support and students’ psychological well-being, yielding valuable insights into this pivotal relationship. Guo et al. (2020) focused their attention on Chinese adolescents and observed that teacher support had a significant impact on mental well-being. This influence was further mediated by the presence of negative emotions and the presence of resilience, underscoring the intricate interplay between teacher support, emotional experiences, and resilience in shaping students’ psychological well-being. Neufeld and Malin (2020) delved into the perceptions of medical students regarding instructor autonomy-support and how these perceptions acted as mediators for their motivation and psychological well-being. Their study illuminated the central role played by instructor autonomy-support in enhancing students’ motivation and overall well-being, emphasizing the importance of fostering supportive teaching approaches to foster positive psychological outcomes.

In a different perspective, Lin et al. (2022) explored the degree of closeness in student-teacher relationships and its influence on the psychological well-being of students. Hope emerged as a mediating factor, with their findings accentuating the significance of robust student-teacher bonds in promoting hope, thereby contributing to heightened psychological well-being.

Virtanen et al. (2019) delved into the alterations in students’ psychological well-being as they transitioned from primary school to lower secondary school. Employing a person-centered approach, they observed varying well-being trajectories during this period, providing valuable insights into the challenges and opportunities encountered by students in distinct well-being trajectories. Furthermore, Van Ryzin et al. (2009) explored the elements of autonomy, a sense of belonging, and engagement within the school environment and their roles in enhancing adolescent psychological well-being. Their research underscored the importance of fostering autonomy, cultivating a sense of belonging, and promoting student engagement within the school as fundamental elements for elevating the psychological well-being of adolescents.

These studies collectively advance our comprehension of how teacher support shapes the psychological well-being of students, unraveling the multifaceted mechanisms involving emotional experiences, resilience, motivation, hope, and school-related factors.

### 2.5 The hypothesized model

This study aims to investigate the interplay between academic self-efficacy, grit, teacher support, and the psychological well-being of Chinese EFL students. Four hypotheses are proposed to elucidate these relationships, grounded in existing research and theories:

*H1*: Academic self-efficacy is directly related to the psychological well-being of EFL students.

This hypothesis is rooted in Bandura’s (1986) social cognitive theory, which posits that an individual’s belief in their capacity to navigate educational tasks and attain academic success, commonly referred to as academic self-efficacy, plays a pivotal role in influencing various aspects of their academic and psychological well-being (Pajares, 1996; Zimmerman, 2000). Previous research has consistently demonstrated a positive association between academic self-efficacy and academic performance, engagement, and psychological well-being (Salami, 2010; Costa et al., 2013; Siddiqui, 2015; Honicke and Broadbent, 2016; Huang, 2016; García-Álvarez et al., 2021; Matteucci and Soncini, 2021; Hong et al., 2022). Students who possess higher levels of academic self-efficacy tend to exhibit reduced anxiety, increased motivation, and a proactive problem-solving orientation, which collectively contribute to their overall psychological well-being (Schwarzer and Jerusalem, 1995). Thus, it is hypothesized that academic self-efficacy directly impacts the psychological well-being of EFL students.

*H2*: Grit is directly associated with the psychological well-being of EFL students.

Recent research also highlights the positive correlation between grit and academic performance, self-confidence, engagement, and motivation (Vainio and Daukantaitė, 2016; Vinothkumar and Prasad, 2016; Arya and Lal, 2018; Huo, 2022). However, the relationship between grit and psychological well-being remains an underexplored area, though it is reasonable to posit that individuals with higher levels of grit may exhibit increased resilience, positive emotional experiences, and overall psychological well-being due to their ability to persevere through adversity (Duckworth, 2016; Fathi and Hejazi, 2023). Therefore, it is hypothesized that grit is directly associated with the psychological well-being of EFL students.

*H3*: Teacher support mediates the relationship between academic self-efficacy and the psychological well-being of EFL students.

Academic self-efficacy, as posited by Bandura’s (1986) social cognitive theory, is foundational in students’ beliefs about their capabilities to succeed academically (Pajares, 1996). Bandura’s theory suggests that individuals’ self-efficacy beliefs influence their behaviors, motivation, and achievements (Bandura, 1997). In the context of education, academic self-efficacy involves students’ perceptions of their ability to effectively engage with academic tasks and achieve desired outcomes (Zimmerman, 2000; Honicke and Broadbent, 2016).

Empirical studies have consistently demonstrated that academic self-efficacy influences individuals’ seeking of social support, particularly from sources such as teachers (Wentzel, 1998; Ryan et al., 2005; Lundberg et al., 2008; Li and Yang, 2009). Students with higher academic self-efficacy are more likely to actively seek and perceive greater support from their teachers (Ryan et al., 2005; Honicke and Broadbent, 2016). This inclination stems from the belief that seeking support from a teacher—a source of expertise and guidance—can aid in enhancing their competence and performance (Huang, 2016; Roorda et al., 2017).

Furthermore, academic self-efficacy is intertwined with students’ willingness to engage in learning activities, and it influences their openness to accepting assistance and feedback, especially from authoritative figures like teachers (Pajares, 1996; Artino, 2012). Students with higher academic self-efficacy tend to interpret teacher support as reinforcing their beliefs in their abilities and fostering an environment conducive to their academic growth (Wentzel, 1998; Mercer et al., 2011; Liu et al., 2018).

In the meantime, previous studies have also suggested that teacher support can enhance students’ overall psychological health by creating trust, reducing anxiety, and promoting a positive learning atmosphere (Van Ryzin et al., 2009; Yang et al., 2018; Virtanen et al., 2019; Guo et al., 2020; Neufeld and Malin, 2020; Lin et al., 2022). Therefore, it is hypothesized that teacher support acts as a mediator in the relationship between academic self-efficacy and the psychological well-being of EFL students.

*H4*: Teacher support mediates the relationship between grit and the psychological well-being of EFL students.

Similar to academic self-efficacy, the relationship between grit and psychological well-being may be mediated by teacher support. Teacher support has been associated with students’ engagement, motivation, and overall well-being (Van Ryzin et al., 2009; Vollet et al., 2017; Guo et al., 2020; Neufeld and Malin, 2020). As grit involves persistence and resilience in the face of challenges, teacher support may play a crucial role in nurturing these qualities, thereby influencing students’ psychological well-being. By creating a supportive and understanding environment, teachers can contribute to reducing anxiety and enhancing students’ overall well-being (Walton and Cohen, 2007). Consequently, it is hypothesized that teacher support acts as a mediator in the relationship between grit and the psychological well-being of EFL students.

## 3 Materials and methods

### 3.1 Participants and procedures

A total of 968 Chinese EFL students from three prominent universities in Shanghai, China, participated in this study. The participants’ age ranged from 18 to 23 years, with a mean age of 20.5 years (SD = 1.26). The sample was well-balanced in terms of gender, with 54.3% male (*n* = 525) and 45.7% female (*n* = 443) participants.

To ensure a diverse representation, students from various academic disciplines were included in the study, with 32% majoring in humanities, 42% in natural sciences, and 26% in social sciences. Moreover, geographical diversity was accounted for, as participants were sourced from different regions across China.

The selection of this sample size was in line with established guidelines within educational research, aiming for an adequate participant-to-variable ratio of at least 10:1 for SEM analyses (Kline, 2016). Inclusion criteria encompassed undergraduate students actively engaged in EFL courses during the current academic term, who willingly volunteered to participate. No specific exclusion criteria were set, except for cases where participants provided incomplete or inconsistent responses within the survey instruments.

Data collection was conducted over a three-month period, from February to April 2022. Informed consent was obtained from each participant after a detailed explanation of the research objectives and procedures. Participants completed structured online questionnaires comprising validated instruments to measure academic self-efficacy, grit, teacher support, and psychological well-being. Each participant was allocated approximately 30 min to ensure thoughtful and accurate responses.

Stringent measures were taken to ensure participant anonymity and confidentiality throughout the data collection process. Personal information remained separate from the collected data, accessible only to authorized research team members. Adherence to ethical guidelines and rigorous data collection procedures were instrumental in maintaining the integrity and reliability of the findings.

### 3.2 Instruments

#### 3.2.1 Academic self-efficacy

To gauge academic self-efficacy, we employed the Academic Self-Efficacy Subscale from the Motivated Strategies for Learning Questionnaire (MSLQ) originally developed by Pintrich and De Groot (1990). This subscale comprises items that capture students’ beliefs in their capabilities to perform well academically. Two sample items included: “I have confidence in my ability to excel in assignments and tests” and “I expect to do well in this class.” Responses were provided on a Likert-style scale, ranging from 1 (indicating “not at all true of me”) to 5 (indicating “very true of me”).

To ensure cultural and linguistic alignment with the Chinese context, a meticulous translation and back-translation process was undertaken, culminating in the creation of the Chinese version of the MSLQ. The resulting scores from individual items were averaged and standardized to establish a comprehensive score reflective of academic self-efficacy, which was integrated into the statistical model. The reliability of the entire scale in this study, estimated by Cronbach’s alpha, was α = 0.88, mirroring the high internal consistency and reliability reported by Pintrich and De Groot (1990) in the original study (Cronbach α = 0.89).

#### 3.2.2 Psychological well-being

Psychological well-being was assessed using the eight-item Flourishing Scale, also known as the PWB (Diener et al., 2010). The translated version of this scale was utilized, which has previously demonstrated high reliability (α = 0.91) and validity among undergraduate and postgraduate students (Lin, 2015). This scale comprised eight items, such as “I lead a purposeful and meaningful life,” where participants provided responses on a 7-point Likert-type scale. On this scale, 1 indicated “strongly disagree” and 7 indicated “strongly agree.”

Additionally, the original study by Diener et al. (2010) reported robust psychometric properties for the Flourishing Scale. Through a principal axis factor analysis, it was revealed that one strong factor, characterized by an eigenvalue of 4.24, accounted for 53 percent of the variance in the items. No other eigenvalue surpassed 1.0, and the factor loadings ranged from 0.61 to 0.77, consolidating the scale’s unidimensional structure and underlining its robustness. The internal consistency of this scale in our study yielded a Cronbach’s alpha of α = 0.89, reaffirming its reliability in measuring psychological well-being among Chinese EFL students.

#### 3.2.3 Grit

Levels of grit were measured using the Short Form Grit Scale (Duckworth and Quinn, 2009). This eight-item scale assesses characteristics related to grit, with statements like “I start whatever I begin.” Participants indicated their level of agreement with each statement on a 5-point Likert scale. On this scale, 1 denoted “not at all like me” and 5 indicated “very much like me.” The original study by Duckworth and Quinn (2009) reported robust findings concerning construct validity. Fit indexes for the Grit–S suggested a strong fit (*χ*^2^ = 106.36, *p* < 0.001; RMSEA = 0.061 [90% CI = 0.050–0.073], CFI = 0.95), consolidating the scale’s validity and supporting its suitability for assessing grit among individuals. In our study, the Short Form Grit Scale exhibited high internal consistency (α = 0.87), denoting reliability in capturing grit levels among the participants.

#### 3.2.4 Teacher support

In this investigation, we assessed the perception of support from educators using the 12-question teacher subcomponent of the Child and Adolescent Social Support Scale. This scale was originally conceived by Malecki and Demary (2002). The teacher subcomponent deconstructs the concept of teacher support into four distinct dimensions: emotional, instrumental, appraisal, and informational support. In the context of our research, we customized these questions to measure students’ perceived support from their English language instructors. As an illustration, one of the items from this subcomponent reads, “My English teacher cares about me.” Participants conveyed their responses using a Likert scale consisting of five options, ranging from “strongly disagree” (1) to “strongly agree” (5). It is worth noting that this scale has demonstrated strong and reliable psychometric characteristics in previous studies (Tennant et al., 2015). The original study conducted by Malecki and Demary (2002) reported robust psychometric properties for this scale, demonstrating an overall Cronbach’s alpha of α = 0.97, indicating an excellent level of internal consistency. Furthermore, the validity assessment in our study yielded favorable results: *χ*^2^ = 2893.39, *df* = 1,480, *χ*^2^/df = 1.95, *p* < 0.001, CFI = 0.99, GFI = 0.99, RMSEA = 0.02, and SRMR = 0.03. In our study, the scale exhibited robust reliability, showcasing a Cronbach’s alpha value of α = 0.92. This score reflects a high level of internal consistency and reliability when gauging Chinese EFL students’ perceptions regarding teacher support.

### 3.3 Data analysis

The data analysis encompassed a methodical exploration of relationships between variables to test the research hypotheses. Initially, SPSS 28.0 was employed to compute descriptive statistics and correlation coefficients, providing a comprehensive overview of the collected data. Given the ordinal nature of the collected data, a transformation into interval data was undertaken while preserving the inherent ordinality of the scale (Cliff, 2014). This transformation ensured compatibility with the statistical methods employed while respecting the original scale’s ordinal properties.

Subsequently, confirmatory factor analysis (CFA) was utilized to assess construct validity and model adequacy using AMOS 26.0. CFA aimed to evaluate the relationships between latent constructs and observed indicators, offering insights into the model’s fit with the data. Further analysis was conducted using SEM, allowing for a comprehensive examination of the intricate relationships among variables within the proposed theoretical framework. To evaluate the goodness of fit, established fit indices were utilized based on the guidelines of Hu and Bentler (1999). These indices included the ratio of *χ*^2^ goodness of fit to the degrees of freedom (df), the Comparative Fit Index (CFI), the Tucker-Lewis Index (TLI), the Root-Mean-Square Error of Approximation (RMSEA), and the Standardized Root-Mean-Square Residual (SRMR). A *χ*^2^/*df* ratio below 3, coupled with a value of *p* exceeding 0.05, indicated a favorable fit. GFI and CFI values equal to or exceeding 0.90 were indicative of a strong fit, while RMSEA values below 0.08 and SRMR values below 0.10 were considered acceptable. To explore the significance of indirect effects, bootstrapping analyses were conducted with 5,000 resamples, following the methodology outlined by Hayes (2009).

## 4 Results

Before commencing the analysis of the proposed model, a comprehensive data screening process was conducted in accordance with established procedures (Tabachnick and Fidell, 2007). This screening involved three key aspects: addressing missing data, assessing normality, and detecting outliers. Each of these components is elaborated upon below.

The presence of missing data is a common challenge in research, and appropriate handling is imperative. Several techniques are available for managing missing data, including list-wise deletion, pair-wise deletion, and the Expectation–Maximization (EM) algorithm (Kline, 2016). Notably, list-wise and pair-wise deletion may not be suitable for studies with a limited sample size, particularly when dealing with a high number of missing data points. In this study, we opted for the EM imputation technique to address missing data. The EM algorithm replaces missing data points with estimated values, enhancing the integrity of the dataset.

To ensure the validity of the data, rigorous normality checks were performed on the collected items utilizing skewness and kurtosis indices. Any values surpassing the established threshold of ±2.0 were deemed indicative of departure from a normal distribution. Such identified values were subsequently omitted from the dataset, aiming to enhance data quality and reliability by addressing non-normally distributed data points. Furthermore, both univariate and multivariate outliers were assessed utilizing standardized Z-scores and the Mahalanobis D^2^ statistic, respectively, as recommended by Tabachnick and Fidell (2007). Identified outliers, recognized as potential influential data points, were consequently removed from the dataset. In total, 8 cases were removed, maintaining the integrity of the analyses and minimizing undue influence from data points significantly deviating from the overall distribution.

Following a meticulous data screening process, CFA was employed to rigorously assess the construct validity of our measurement models. This critical step aimed to ascertain the appropriateness of our models and was guided by an examination of goodness-of-fit indices. Our CFA analysis involved the assessment of measurement models for all latent constructs, encompassing academic self-efficacy, grit, teacher support, and psychological well-being. During the initial analyses, some of these measurement models did not exhibit a strong fit with the data. Consequently, necessary modifications were applied to align the models more closely with the observed data patterns.

To ensure robust alignment between the model and empirical data, critical adjustments were executed. Initial scrutiny revealed that two items from the grit scale, two items from the psychological well-being scale, and one item from the academic self-efficacy scale demonstrated factor loadings below the threshold of 0.40. The decision to exclude these items was predicated on the premise that items failing to meet this threshold might not adequately contribute to the measurement of their respective constructs (Hancock and Mueller, 2001; Hair et al., 2014).

Theoretical and empirical justifications underpin this decision. Theoretical constructs are best captured through items that manifest strong factor loadings, ensuring their substantive contribution to the overall construct (Fornell and Larcker, 1981). Consequently, items falling below the threshold were considered less representative or potentially ambiguous in measuring the intended constructs. This aligns with the premise that factor loadings below 0.40 might signify weak relationships between the items and their latent constructs, raising concerns about their conceptual alignment (Hair et al., 2014).

Additionally, to address model-data discrepancies, two correlational paths were introduced between error terms of two latent constructs. This strategic adjustment accounted for potential shared variance or omitted variables, thus refining the model’s accuracy and robustness in explaining the observed data patterns (Kline, 2016).

These methodological adjustments were pivotal in refining the measurement models, leading to a more precise representation of the latent constructs within the empirical context. The revised models displayed commendable fit indices, as meticulously detailed in Table 1, reinforcing the alignment between the theoretical constructs and empirical data.

**Table 1:** The results of CFA.

	*χ* ^2^	*df*	*χ*^2^/*df*	CFI	TLI	RMSEA	SRMR
Self-efficacy	101.73	51	1.99	0.922	0.918	0.051	0.065
Grit	94.72	48	1.97	0.934	0.926	0.046	0.062
Teacher support	172.13	96	1.79	0.981	0.972	0.024	0.035
Well-being	216.36	114	1.89	0.973	0.969	0.033	0.044

Additionally, we conducted a sequence of confirmatory factor analyses to assess the unidimensionality of the latent factors. Three alternative measurement models were juxtaposed against the hypothesized baseline model for comparison. The findings, detailed in Table 2, demonstrate that among the models tested, the initially hypothesized four-factor measurement model exhibited a superior fit to the data. This model displayed favorable indices: *χ*^2^ = 720.901, *df* = 507, *p* < 0.001, CFI = 0.979, TLI = 0.944, RMSEA = 0.027, and SRMR = 0.063. Subsequently, we calculated descriptive statistics and inter-variable correlations as seen in Table 3.

**Table 2:** Comparative fit indices of measurement models.

Measurement model	*χ* ^2^	*df*	CFI	TLI	RMSEA	SRMR
Single-factor model	853.789	511	0.956	0.921	0.038	0.205
Two-factor model	819.491	509	0.959	0.926	0.036	0.178
Three-factor model	778.269	508	0.967	0.937	0.032	0.125
Four-factor model	720.901	507	0.979	0.944	0.027	0.063

**Table 3:** Descriptive statistics and correlations.

	M (SD)	1	2	3	4
1. Self-efficacy	3.16 (0.53)	1.00			
2. Grit	2.67 (0.66)	0.25^*^	1.00		
3. Teacher support	2.79 (0.71)	0.32^**^	0.43^**^	1.00	
4. Well-being	3.09 (0.59)	0.51^**^	0.36^**^	0.47^**^	1.00

^*^p < 0.05. ^**^p < 0.01.

In Table 3, we present the descriptive statistics and correlations among the variables. Self-efficacy was found to have a mean of 3.16 (SD = 0.53), indicating a moderate level of self-belief in handling educational tasks. Grit exhibited a mean of 2.67 (SD = 0.66), reflecting the level of perseverance and determination among the participants. Teacher support had a mean of 2.79 (SD = 0.71), indicating the perceived level of support from educators. Lastly, well-being showed a mean of 3.09 (SD = 0.59), reflecting the reported psychological well-being of the participants. The correlations between these variables were also examined. As expected, the relationships between variables, grit showed a positive correlation with self-efficacy (*r* = 0.25, *p* < 0.05), indicating that as students’ self-efficacy increases, so does their level of grit.

Furthermore, teacher support displayed significant and positive correlations with both self-efficacy (*r* = 0.32, *p* < 0.01) and grit (*r* = 0.43, *p* < 0.01). This implies that students who receive more teacher support tend to have higher levels of self-efficacy and grit. Finally, well-being exhibited the highest positive correlations with all other variables. It was significantly related to self-efficacy (*r* = 0.51, *p* < 0.01), grit (*r* = 0.36, *p* < 0.01), and teacher support (*r* = 0.47, *p* < 0.01). This suggests that students with higher levels of self-efficacy, grit, and teacher support reported greater psychological well-being.

In addition, an exploration of gender-based variations in the four constructs was conducted through independent-samples t-tests. Notably, these analyses revealed that there were no statistically significant differences between males and females with respect to the four examined constructs. This finding underscores the absence of gender-related disparities in our study’s variables.

In Table 4, we rigorously evaluate the convergent and divergent validity of our latent variables, employing average variance extracted (AVE) and the square root of AVE as assessment metrics, aligning with the principles outlined by Fornell and Larcker (1981).

**Table 4:** Assessment of convergent and divergent validity.

	AVE	Square roots
Self-efficacy	0.65	0.81
Grit	0.57	0.75
Teacher support	0.63	0.79
Well-being	0.71	0.84

AVE measures the average variance explained by each latent variable’s indicator variables. Convergent validity is confirmed when AVE exceeds 0.50 (50%), indicating a strong association with its indicators. As demonstrated in the table, all latent variables, encompassing self-efficacy (AVE = 0.65), grit (AVE = 0.57), teacher support (AVE = 0.63), and well-being (AVE = 0.71), surpass this threshold, affirming their robust convergent validity.

Divergent validity is confirmed when the AVE of a latent variable surpasses the squared correlations with other latent constructs. In this study, the AVE values are expectedly higher than the squared correlations, indicating a strong presence of robust divergent validity among the latent variables.

SEM was employed to rigorously investigate the proposed relationships among self-efficacy and grit serve as direct predictors of psychological well-being, while teacher support was the mediator in this model. The assessment of model fit indices confirmed that the model aligned well with the observed data, indicating that it was a satisfactory representation of the relationships within our study. Specifically, the results of the model fit indices are as follows: *χ*^2^ (421) = 650.79, *p* = 0.000, CFI = 0.978, TLI = 0.974, RMSEA = 0.041, 95% CI = 0.037–0.043.

**Figure 1:**
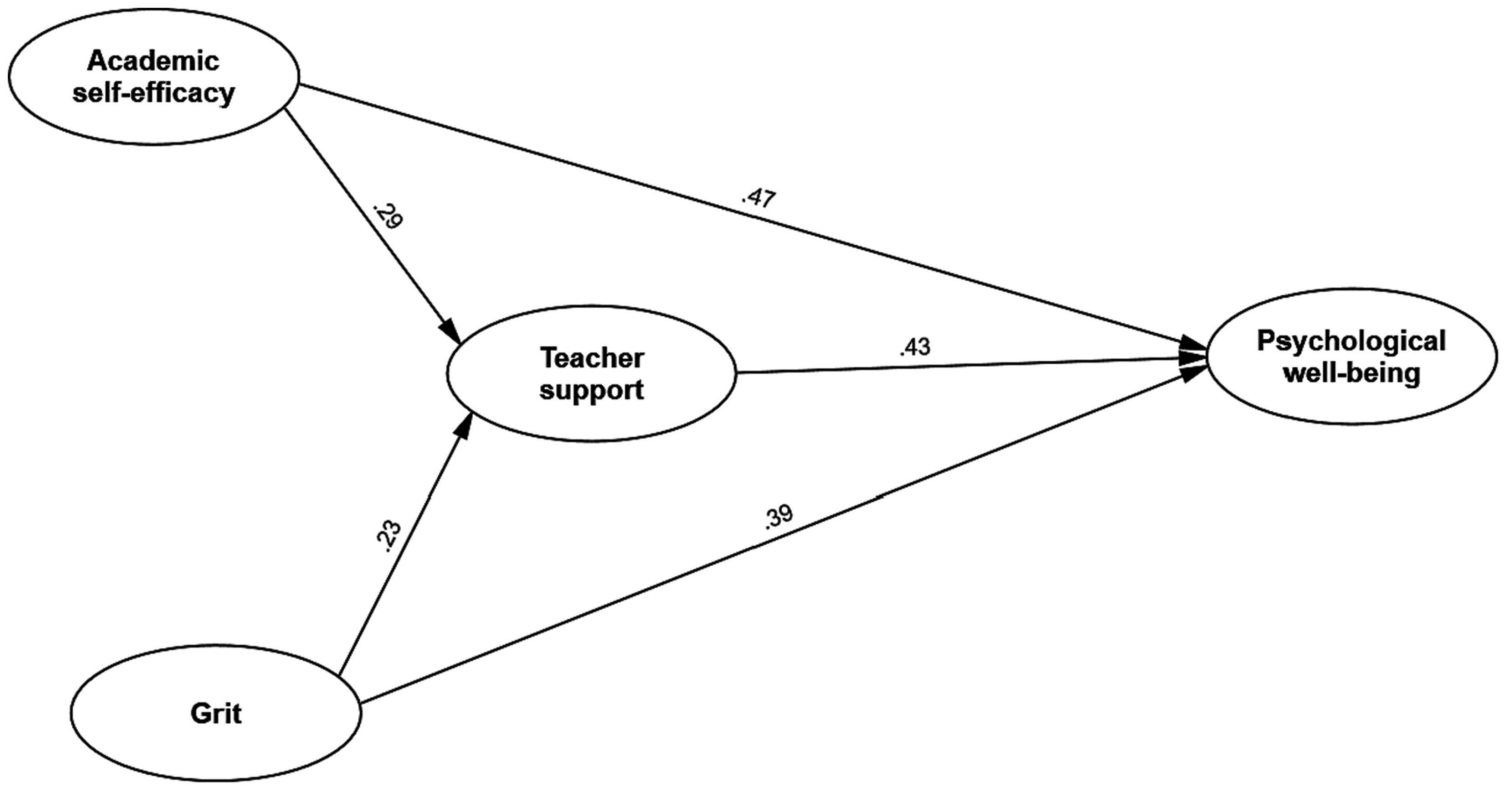
The final model.

To visually represent these relationships, Figure 1 illustrates the path diagram, displaying the interconnectedness of the latent constructs. All path coefficients in the model were determined to be statistically significant, thereby substantiating the anticipated associations between the variables under investigation.

In particular, our analysis revealed several key findings. Firstly, academic self-efficacy displayed a positive and statistically significant relationship with well-being (*β* = 0.47). Additionally, teacher support exhibited a positive connection with well-being (*β* = 0.43), as did grit (*β* = 0.39). Furthermore, academic self-efficacy showed a notable influence on well-being (*β* = 0.29), emphasizing its relevance in fostering a sense of psychological well-being. Lastly, the relationship between grit and teacher support was found to be significant (*β* = 0.23), highlighting the interplay between these two constructs within our study.

Finally, to assess the significance of the indirect effects, bootstrapping analyses were conducted using 5,000 resamples (Hayes, 2009). The mediation analysis revealed a series of significant direct and indirect effects, as summarized in Table 5.

**Table 5:** Direct, indirect, and total effects.

Path	Coefficient (*β*)	Standard error (SE)	*p*	Effect type
Self-efficacy → well-being	0.47	0.04	<0.001	Direct
Teacher support → well-being	0.43	0.05	<0.001	Direct
Grit → well-being	0.39	0.05	<0.001	Direct
Self-efficacy → teacher support → well-being	0.12	0.03	<0.01	Indirect
Grit → teacher support → well-being	0.09	0.03	<0.01	Indirect
Self-efficacy → well-being	0.59	0.04	<0.001	Total
Grit → well-being	0.48	0.05	<0.001	Total

Table 5 presents the direct, indirect, and total effects in the mediation analysis. Firstly, we observed that academic self-efficacy had a significant direct effect on well-being (*β* = 0.47, SE = 0.04, *p* < 0.001). Similarly, teacher support exhibited a substantial direct effect on well-being (*β* = 0.43, SE = 0.05, *p* < 0.001), as did grit (*β* = 0.39, SE = 0.05, *p* < 0.001).

Intriguingly, the mediation analysis also unveiled important indirect effects. The path from academic self-efficacy to well-being through teacher support was found to be significant (*β* = 0.12, SE = 0.03, *p* < 0.01). Likewise, the indirect pathway from grit to well-being through teacher support also yielded a significant effect (*β* = 0.09, SE = 0.03, *p* < 0.01).

In summary, the total effects of academic self-efficacy and grit on well-being were notably significant (*β* = 0.59, SE = 0.04, *p* < 0.001 and *β* = 0.48, SE = 0.05, *p* < 0.001, respectively). These results emphasize the complex interplay of these variables and their combined influence on student well-being within the educational context.

## 5 Discussion

The objective of this study was to explore the associations between academic self-efficacy, grit, teacher support, and the psychological well-being of EFL students, aiming to provide valuable insights into how these factors are interconnected and how they collectively influence the overall well-being of language learners.

First, it was found that academic self-efficacy was directly related to the psychological well-being of EFL learners. This outcome aligns consistently with a wealth of existing research, establishing a strong connection between self-efficacy and psychological well-being (Salami, 2010; Costa et al., 2013; Siddiqui, 2015; García-Álvarez et al., 2021; Matteucci and Soncini, 2021; Hong et al., 2022). Individuals who hold higher self-efficacy beliefs exhibit a propensity to set ambitious goals, persist in the face of adversity, and demonstrate effective resilience when encountering setbacks (Hwang et al., 2016; Jung et al., 2017; Lee and Seo, 2021). These attributes share a significant association with psychological well-being. Students with elevated academic self-efficacy commonly report experiencing reduced levels of stress and anxiety, heightened motivation, and an enhanced sense of control over their learning experiences (Pajares, 1996; Diseth et al., 2012; Hoigaard et al., 2015; Zimmerman and Kulikowich, 2016; Musa, 2020).

In the context of EFL learners, the role of academic self-efficacy takes on particular significance. Learning a new language can indeed be a daunting and challenging task (Genc et al., 2016). However, individuals equipped with a strong sense of academic self-efficacy approach language acquisition with a positive and optimistic mindset (Shang, 2010). These learners perceive language acquisition as a conquerable task and, notably, exhibit greater resilience when confronted with language-related difficulties (Hsieh and Kang, 2010).

Moreover, the linkage between academic self-efficacy and self-regulation, a fundamental aspect of successful language learning (Zimmerman et al., 1992; Zimmerman, 2002), is a crucial one. EFL learners with high academic self-efficacy demonstrate advanced skills in goal-setting, effective planning of learning strategies, and a remarkable capacity to persevere through challenges. These attributes contribute significantly to the enhancement of psychological well-being (Salami, 2010; García-Álvarez et al., 2021; Matteucci and Soncini, 2021). Consequently, these learners are more likely to experience a profound sense of accomplishment, a fundamental component of psychological well-being in the educational context (Diener et al., 2009; Diseth, 2011). Importantly, it is essential to recognize that the relationship between academic self-efficacy and psychological well-being is not unidirectional. As learners experience improved psychological well-being, their self-efficacy beliefs may, in turn, be further fortified. This dynamic interaction between self-efficacy and well-being underscores the pivotal role of academic self-efficacy in shaping the overall educational experience of EFL learners (Jerusalem and Schwarzer, 2014).

Second, it was revealed that grit was directly associated with the psychological well-being of EFL students. This result is in alignment with a significant body of existing literature (Vainio and Daukantaitė, 2016; Vinothkumar and Prasad, 2016; Arya and Lal, 2018; Huo, 2022) and significantly contributes to a deeper understanding of the role of perseverance and passion for long-term goals in the context of EFL education. The association between grit and psychological well-being can be theoretically understood through the lens of Positive Psychology which emphasizes the cultivation of character strengths and resilience as fundamental elements to enhance overall well-being (Seligman and Csikszentmihalyi, 2000). Grit, with its emphasis on passion and perseverance, closely aligns with the principles of character strengths and resilience. Students who exhibit grit are more likely to face the challenges of language learning with resilience and adaptability, which, in turn, can lead to improved psychological well-being (Hu et al., 2022; Teimouri et al., 2022).

Numerous studies have explored the role of grit in various educational contexts and consistently reported positive associations with student achievement and psychological well-being (Vainio and Daukantaitė, 2016; Datu et al., 2018; Huo, 2022). From this perspective, research indicates that grit significantly contributes to long-term language proficiency development among students (Datu et al., 2018; Alamer, 2021). Gritty language learners tend to persist through language difficulties, maintain their motivation over time, and exhibit increased levels of self-regulation. These factors are conducive to enhanced psychological well-being (Khajavy et al., 2021; Pawlak et al., 2022).

Grit is intrinsically connected to the concept of goal-setting and achievement. EFL students with high levels of grit set ambitious language learning goals and demonstrate a strong willingness to invest time and effort in achieving those goals (Duckworth et al., 2007; Wei et al., 2020). The achievement of these language learning objectives significantly contributes to feelings of accomplishment, which are integral to psychological well-being in the context of education (Zhou, 2023).

Furthermore, grit has been associated with lower levels of stress and anxiety (Musumari et al., 2018). Language learning can be a potential source of anxiety for many EFL students, especially when confronted with challenges like speaking in a foreign language or comprehending complex grammar rules (Liu, 2022). Gritty individuals tend to perceive these challenges as opportunities for growth rather than as threats. This perspective can lead to a reduction in anxiety and, consequently, an enhancement of psychological well-being (Zhao and Wang, 2023).

Third, teacher support mediated the relationship between academic self-efficacy and psychological well-being of EFL students. This result resonates with existing literature that underscores the significance of teacher support in shaping students’ motivation, learning behaviors, and academic performance (Pajares, 1996; Jung et al., 2017; Musa, 2020). Academic self-efficacy stands as a pivotal factor in determining students’ educational journey. It empowers them to set ambitious goals, persist in the face of challenges, and experience reduced levels of anxiety, ultimately contributing to enhanced psychological well-being (Bandura, 1986; Salami, 2010; Costa et al., 2013; Siddiqui, 2015; García-Álvarez et al., 2021; Matteucci and Soncini, 2021; Hong et al., 2022). In environments where students perceive their teachers as supportive, encouraging, and responsive, there is an increase in their sense of autonomy and competence, which in turn leads to elevated motivation and overall well-being (Ryan and Deci, 2000; Oriol-Granado et al., 2017). This is particularly pertinent in the realm of EFL education, where language learning can be a challenging endeavor.

The mediating role of teacher support aligns seamlessly with broader research on teacher-student relationships. Favorable teacher-student relationships have been associated with a range of positive outcomes, including heightened engagement, motivation, and overall well-being (Fraser and Walberg, 2005; Roorda et al., 2011; Wubbels et al., 2016). In case learners perceive their instructors as supportive, it not only bolsters their confidence in their academic abilities but also fosters a positive emotional climate in the classroom, conducive to improved psychological well-being (Schunk and Pajares, 2009; Zee and Koomen, 2016). Furthermore, teacher support serves as a crucial buffer against the distinctive obstacles encountered by EFL students, including linguistic hindrances and adaptations to diverse cultures. Encouragement and support from teachers equip students to navigate these obstacles effectively, resulting in reduced levels of stress and anxiety, ultimately contributing to enhanced psychological well-being (Yang, 2021).

Finally, it was found that teacher support mediated the relationship between grit and the psychological well-being of EFL students. Gritty individuals exhibit remarkable resilience and determination in the pursuit of their objectives, attributes closely associated with positive well-being (Duckworth and Gross, 2014). Their ability to overcome challenges and setbacks is a testament to the profound influence of grit on one’s psychological disposition.

Teacher support emerges as a pivotal factor in fostering student motivation, engagement, and overall well-being. Educators who provide autonomy support, positive feedback, and cultivate a nurturing learning environment wield significant influence over their students’ motivation, self-efficacy, and overall well-being (Ryan and Deci, 2000; Alivernini and Lucidi, 2011; Aldridge et al., 2013). This influence is particularly pronounced in the context of EFL education, where language learning can be an inherently daunting and challenging process. The mediating role of teacher support aligns seamlessly with broader research on teacher-student relationships and their impact on well-being. Positive teacher-student relationships have consistently demonstrated associations with increased student engagement, motivation, and overall well-being (Wubbels et al., 2016). When students perceive their teachers as supportive, it not only bolsters their confidence in their ability to surmount challenges but also fosters a positive emotional climate in the classroom, thereby contributing to improved psychological well-being (Kim et al., 2018).

In the process of learning a foreign language, EFL students frequently encounter difficulties in comprehension, speaking, and cultural adaptation. Gritty students, driven by their determination and passion for long-term goals, exhibit a greater propensity to persist through these challenges (Eskreis-Winkler et al., 2014). Here, teacher support emerges as a critical facilitator, providing guidance, encouragement, and the emotional support necessary to sustain this perseverance. This, in turn, significantly contributes to enhanced psychological well-being (Tennant et al., 2015; Brandseth et al., 2019).

## 6 Implications for theory and practice

This research study has unveiled the complex interplay among academic self-efficacy, grit, teacher support, and the psychological well-being of Chinese EFL students, offering significant implications for both educational practitioners and policymakers. The study has unequivocally emphasized the fundamental roles these constructs hold within the realm of EFL education.

The findings vividly underscore the pivotal role of academic self-efficacy in shaping the psychological well-being of EFL learners. Educational contexts play a crucial role in fostering and nurturing self-efficacy beliefs among students. Self-efficacy, as Bandura (1977) proposed, is not a fixed trait; it evolves through mastery experiences, social modeling, social persuasion, and interpretations of physiological feedback. Therefore, educational institutions could design targeted interventions to enhance self-efficacy by providing diverse opportunities for students to succeed in challenging academic tasks, offering encouragement and constructive feedback, and guiding students to identify and acknowledge their strengths (Bandura, 1982).

Similarly, promoting grit among students involves cultivating perseverance and passion towards long-term goals. Training programs within educational settings can be devised to instill the principles of grit, such as emphasizing the importance of sustained effort and maintaining resilience in the face of obstacles (Duckworth et al., 2007). In addition, providing mentorship, setting achievable yet challenging goals, and highlighting the value of perseverance could significantly contribute to the development of grit among EFL learners.

Understanding how self-efficacy develops is pivotal (Zammitti et al., 2023). Self-efficacy beliefs, as proposed by Bandura (1986), stem from various sources including mastery experiences, social modeling, verbal persuasion, and interpretation of physiological states. Mastery experiences, where students succeed in tasks or overcome challenges, are particularly influential in developing self-efficacy. Educators and policymakers can focus on creating environments that offer a balance of achievable challenges and opportunities for success to reinforce positive mastery experiences. Moreover, fostering a growth mindset that encourages students to view failures as learning opportunities and providing constructive feedback can significantly contribute to the development of academic self-efficacy (Bandura, 1986).

## 7 Limitations and conclusions

While this study provides valuable insights, several limitations should be acknowledged. Firstly, the reliance on self-report measures introduces potential response bias and common method variance. Future research efforts should diversify data sources and methodologies to enhance the robustness and validity of the findings. Additionally, it’s crucial to acknowledge that the sample used in this study was not probabilistic, but rather drawn from specific universities in Shanghai, China. As a result, the non-probabilistic nature of the sample limits the generalizability of the findings beyond this particular context, potentially hindering the broader applicability of the results to other cultural and linguistic settings. Different cultural nuances, educational systems, and language learning environments might impact the relationships among academic self-efficacy, grit, teacher support, and psychological well-being in distinct ways.

Furthermore, the study’s focus on specific mediating relationships might not fully encapsulate the complexity of interactions among these constructs. There could be other unexplored variables that play a role in shaping the relationships observed in this study. Future investigations could delve deeper into additional factors that might influence or mediate the connections among academic self-efficacy, grit, teacher support, and psychological well-being within diverse educational contexts.

In conclusion, this study presents compelling insights into the intricate and interconnected relationships among academic self-efficacy, grit, teacher support, and the psychological well-being of Chinese EFL students. The findings underscore the profound impact of these constructs on students’ experiences within the educational landscape. The established relationships elucidate how academic self-efficacy serves as a cornerstone in shaping the psychological well-being of EFL learners. This fundamental belief in one’s ability to achieve academic success not only influences their attitudes toward learning but also significantly contributes to their overall psychological health. Moreover, the demonstrated significance of grit showcases the enduring impact of perseverance and passion on fostering a positive outlook amid the challenges inherent in language learning. Notably, teacher support emerges as a vital mediator, indicating the crucial role educators play in nurturing a supportive environment conducive to students’ academic self-efficacy and grit. This underscores the immense responsibility of educators in creating classrooms that foster autonomy, offer constructive feedback, and provide emotional encouragement to cultivate students’ well-being.

## Data availability statement

The raw data supporting the conclusions of this article are made available by the authors, without undue reservation. Requests to access these datasets should be directed to LT, tang05311516@sina.cn.

## Ethics statement

The studies involving humans were approved by School of Foreign Languages, Shandong University, Jinan 250100, China. The studies were conducted in accordance with the local legislation and institutional requirements. The participants provided their written informed consent to participate in this study.

## Author contributions

LT: Conceptualization, Data curation, Formal analysis, Funding acquisition, Investigation, Methodology, Project administration, Resources, Supervision, Validation, Visualization, Writing – original draft, Writing – review & editing. XZ: Conceptualization, Data curation, Formal analysis, Investigation, Methodology, Project administration, Software, Validation, Visualization, Writing – original draft, Writing – review & editing.
